# A Royal Chartered College joins *Chiropractic & Manual Therapies*

**DOI:** 10.1186/2045-709X-21-1

**Published:** 2013-01-04

**Authors:** Bruce F Walker, Robert P Finch, Timothy C Jay

**Affiliations:** 1 Chiropractic & Manual Therapies, Murdoch University, Perth, Western Australia, Australia; 2 The College of Chiropractors, Chiltern Chambers, St Peters Avenue, Reading, RG4 7DH, UK

## Abstract

From January 2013, The UK College of Chiropractors joins the partnership between the Chiropractic and Osteopathic College of Australasia (COCA) and the European Academy of Chiropractic (EAC) to publish *Chiropractic & Manual Therapies.* This new partnership will enable the journal to grow and flourish and further improve access to high quality research publications for interested researchers and clinicians worldwide.

## Introduction

From January 2013 The UK College of Chiropractors (TCoC), a professional chiropractic membership body [1], joins the partnership between the Chiropractic and Osteopathic College of Australasia (COCA) [2] and the European Academy of Chiropractic (EAC) [3] to publish *Chiropractic & Manual Therapies.* COCA is an Australian based professional college for chiropractors and osteopaths and the EAC is the academic committee of the European Chiropractors’ Union (ECU) [4]*.* This new partnership will enable the journal to grow and publish more quality research articles relating to chiropractic and other manual therapies. It will provide the three joint venture partners with a world class journal of their own which has the capacity to disseminate knowledge worldwide using the free full text online open access format with BioMed Central as publishers.

### The new partner

Ever since its initial inception as the British College of Chiropractic, the organisation currently known as The College of Chiropractors (TCoC) has produced a professional journal. Volume 1, Issue 1 of The British Journal of Chiropractic was published in September 1997 to meet the *‘need for a professional journal as a means of communication within the profession and to allied disciplines, as a forum for debate and as a focal point for the rich diversity of practitioners in the UK’*[5]. Five volumes and six years later in 2003, and with Elsevier as the publisher, the journal was re-launched as *Clinical Chiropractic*, aimed at an international audience and specifically intended *‘to provide authoritative information of use to the clinical chiropractor in advancement of their professional career, clinical skills and performance, and ability to deliver optimal patient care’*[6]*.* Throughout its 15-year history, the journal has been edited by Martin Young whose understanding and vision of the importance of evidence-based chiropractic practice has shaped every issue, and whose personal dedication to the encouragement of others to publish chiropractic research has been remarkable.

Recently, TCoC has entered a new era as a Royal Charter-incorporated organisation [7]. In the UK, Royal Charters are a rare means of incorporating a body as a single legal entity and may be granted by the Monarch to eminent professional bodies or charities that have a solid record of achievement, are stable and permanent, and for which incorporation by Charter is in the public interest [8]. In this new era, the Royal Chartered college has ceased publication of *Clinical Chiropractic* although all back issues will remain accessible on the Elsevier Science Direct platform. The Royal Chartered college sees the open access nature of *CMT* as reducing the barriers to research literacy and providing the strongest platform from which to publish excellence in chiropractic and manual therapy research, hence the decision to join forces with COCA and the EAC to publish *Chiropractic & Manual Therapies*.

## Conclusion

All of the journal’s joint venture partners and its editorial team are excited about the opportunity to expand the journals offerings to all readers but particularly to members of the three partner organisations and their affiliates. The journal has gone from strength to strength and this new development augurs well for the future.
